# Supplementary material for: Evidence for a novel cranial thermoregulatory pathway in thalattosuchian crocodylomorphs
Source: PeerJ. 2023 May 2;11:e15353. doi: 10.7717/peerj.15353 (PMC10162039; doi:10.7717/peerj.15353)
Supplement: Supplemental Information 1 [file peerj-11-15353-s001.docx]

**Online Supplementary material**

**Supplementary Institutional abbreviations**

**AMNH**, American Museum of Natural History, New York City, New York, USA; **CM**, Carnegie Museum of Natural History, Pittsburgh, Pennsylvania, USA; **FMNH**, Field Museum of Natural History, Chicago, Illinois, USA; **IRSNB**, Institut Royal des Sciences Naturelles de Bruxelles, Belgium; **IVPP**, Institute of Paleontology and Paleoanthropology, Beijing, China; **IWCMS**, Isle of Wight County Museums Services (Dinosaur Isle Museum and visitor attraction) Sandown, United Kingdom; **MLP**, Museo de La Plata, La Plata, Argentina; **MM**, Minden Museum, Minden, Germany; **MNB**, National Museum of the Bahamas, Nassau, Bahamas; **MNHN**, Muséum national dʼHistoire naturelle, Paris, France; **MTM**, Magyar Természettudományi Múzeum, Budapest, Hungary; **NMS**, National Museum of Scotland, Edinburgh, Scotland, UK; **NHMUK**, Natural History Museum, London, UK; **OUVC**, Ohio University, Vertebrate Collection, Athens, Ohio, USA; **TMM**, Texas Memorial Museum, University of Texas at Austin, Austin, Texas, USA; **UF**, University of Florida, Florida Museum of Natural History, Gainesville, Florida, USA; **USNM**, National Museum of Natural History, Washington DC, USA.

**TABLE S1.** List of specimens examined herein.

| **Species** | **Specimen number** | **Age** | **Voxel size (mm)** | **Facility/Source** |
| --- | --- | --- | --- | --- |
| *Alligator mississippiensis* | OUVC 10606 | Recent | 0.045 | Ohio University MicroCT Facility, USA |
| *Alligator mississippiensis* | OUVC 9757 | Recent | 0.09 | Ohio Health O'Bleness Hospital, USA |
| *Alligator mississippiensis* | OUVC 9761 | Recent | 0.5 X 1 | Ohio Health O'Bleness Hospital, USA |
| *Alligator mississippiensis* | OUVC 11415 | Recent | 0.0493 | Ohio University MicroCT Facility, USA |
| *Alligator mississippiensis* | TMM M983 | Recent | 0.25 X 0.48 | High-Resolution X-ray CT facility, University of Texas, USA |
| *Alligator mississippiensis* | USNM 211233 | Recent | 0.625 | Ohio Health O'Bleness Hospital, USA |
| *Caiman crocodilus* | FMNH 73711 | Recent | 0.065 X 0.142 | High-Resolution X-ray CT facility, University of Texas, USA |
| *Crocodylus acutus* | FMNH 59071 | Recent | 0.625 | Ohio Health O’Bleness Hospital, USA |
| *Crocodylus rhombifer* | MNB AB50.0171 | Recent | 0.1748 X 0.5 | High-Resolution X-ray CT facility, University of Texas, USA |
| *Crocodylus moreletii* | TMM M-4980 | Recent | 0.1904 X 0.5 | High-Resolution X-ray CT facility, University of Texas, USA |
| *Crocodylus porosus* | OUVC 10899 | Recent | 0.0472 | Ohio Health O'Bleness Hospital, USA |
| *Osteolaemus tetraspis* | FMNH 98936 | Recent | 0.0546875 X 0.1108 | High-Resolution X-ray CT facility, University of Texas, USA |
| *Crocodylus johnstoni* | TMM M-6807 | Recent | 0.223 | High-Resolution X-ray CT facility, University of Texas, USA |
| *Mecistops cataphractus* | TMM M-3529 | Recent | 0.165 X 0.5 | High-Resolution X-ray CT facility, University of Texas, USA |
| *Gavialis gangeticus* | TMM M-5490 | Recent | 0.228 | High-Resolution X-ray CT facility, University of Texas, USA |
| *Gavialis gangeticus* | UF-herp-118998 | Recent | 0.14654672 | Florida Museum of Natural History, USA |
| *Tomistoma schlegelii* | USNM 211322 | Recent | 0.625 | Ohio Health O'Bleness Hospital, USA |
| *Tomistoma schlegelii* | TMM M-6342 | Recent | 0.165 X 0.46 | High-Resolution X-ray CT facility, University of Texas, USA |
| *Plagiophthalmosuchus gracilirostris* | NHMUK PV OR 15500 | Toarcian | 0.236872 X 0.1185 | µVIS X-Ray Imaging Centre, University of Southampton, UK |
| *Pelagosaurus typus* | NHMUK PV OR 32599 | Toarcian | 0.098627983 | Nikon XT H 225S CT system, Natural History Museum, London, UK |
| *Eoneustes gaudryi* | NHMUK PV R 3263 | Bathonian | 0.159849 | µVIS X-Ray Imaging Centre, University of Southampton, UK |
| *Thalattosuchus superciliosus* | NHMUK PV R 11999 | Callovian | 0.12 | µVIS X-Ray Imaging Centre, University of Southampton, UK |
| *Cricosaurus araucanensis* | MLP 72-IV-7-1 | Tithonian | 0.448 | Hospital Interzonal de Agudos de la Matanza “Dr. Diego Pairoissien” La Matanza, Argentina |
| *Cricosaurus schroederi* | MM Pa1 | Valanginian | 0.5 | Leibniz Institute for Zoo and Wildlife Research, Berlin, Germany |

**TABLE S2.** List of crocodylomorph specimens examined first-hand that lack palatal grooves. Fossil taxa denoted by †.

| **Species** | **Specimen numbers (and literature showing the palate)** | **Taxonomic group** |
| --- | --- | --- |
| *Theriosuchus* *pusillus* † | NHMUK PV OR 48216 | Atoposauridae, Neosuchia |
| *Anteophthalmosuchus hooleyi* † | NHMUK PV R 3876; Ristevski *et al*., 2018 | Goniopholididae, Neosuchia |
| *Anteophthalmosuchus epikrator* † | IWCMS 2001.446, IWCMS 2005.127; Ristevski *et al*., 2018 | Goniopholididae, Neosuchia |
| *Eutretauranosuchus delfsi* † | CM 8028; Pritchard *et al*., 2013 | Goniopholididae, Neosuchia |
| *Pholidosaurus purbeckensis* † | NHMUK PV R 3956, NHMUK PV R 36721 | Pholidosauridae, Neosuchia |
| *Terminonarus browni* † | AMNH 5851 | Pholidosauridae, Neosuchia |
| *Elosuchus broinae* † | MNHN.F SAM 129; Meunier & Larsson, 2017 | Dyrosauroidea, Neosuchia |
| *Elosuchus cherifensis* † | MNHN.F MRS 340; Meunier & Larsson, 2017 | Dyrosauroidea, Neosuchia |
| *Koumpiodontosuchus aprosdokiti* † | IWCMS 2012.203-204; Sweetman *et al*., 2015 | Bernissartiidae, Neosuchia |
| *Iharkutosuchus makadii* † | MTM 2006.52.1; Ősi *et al*., 2007 | Hylaeochampsidae, Eusuchia |
| *Diplocynodon hantoniensis* † | NHMUK PV OR 25166, NHMUK PV OR 30392; Rio *et al*., 2020 | Alligatoroidea, Eusuchia |
| *Alligator mississippiensis* | NHMUK 68.2.12.6, NHMUK ZD 290, NHMUK ZD 1973-2-21-2, NHMUK ZD 1974-3010, NHMUK ZD 1975-1424, NHMUK ZD II-1-I, OUVC 9761, OUVC 10606, OUVC 11415, TMM M983, USNM 211233 | Alligatoridae, Eusuchia |
| *Alligator sinensis* | NHMUK X184 | Alligatoridae, Eusuchia |
| *Caiman crocodilus* | FMNH 73711, NHMUK 1898.9.26.1, NHMUK 1933.5.10.1 | Alligatoridae, Eusuchia |
| *Caiman latirostris* | NHMUK 2008-270, NHMUK 86.10.4.2 | Alligatoridae, Eusuchia |
| *Melanosuchus niger* | NHMUK 1945-8-25-126 | Alligatoridae, Eusuchia |
| *Paleosuchus trigonatus* | NHMUK 1868.10.8.1 | Alligatoridae, Eusuchia |
| *Brochuchus pigotti* † | NHMUK PV R 7729 | Crocodylidae, Eusuchia |
| *Crocodylus acutus* | FMNH 59071, NHMUK 1975.997 | Crocodylidae, Eusuchia |
| *Crocodylus halli* | NHMUK 1886.5.20.1, NHMUK 1886.5.20 | Crocodylidae, Eusuchia |
| *Crocodylus intermedius* | NMS Z.1945.42 | Crocodylidae, Eusuchia |
| *Crocodylus johnstoni* | TMM M-6807 | Crocodylidae, Eusuchia |
| *Crocodylus moreletii* | TMM M-4980, NHMUK 1861.4.14 | Crocodylidae, Eusuchia |
| *Crocodylus niloticus* | NHMUK 1949-1-1-2, NHMUK 1959.1.8.55 | Crocodylidae, Eusuchia |
| *Crocodylus palaeindicus* † | NHMUK PV OR 39795 | Crocodylidae, Eusuchia |
| *Crocodylus palustris* | NHMUK 1868.4.9.11, NMS Z.1945.43 | Crocodylidae, Eusuchia |
| *Crocodylus porosus* | NHMUK 1847.3.5.33, NHMUK 1929-2-225-3803, NHMUK 1943-8-18-4, NHMUK 1947-3-5-33, OUVC 10899 | Crocodylidae, Eusuchia |
| *Crocodylus rhombifer* | MNB AB50.0171, NMS Z.2014.18.2 | Crocodylidae, Eusuchia |
| *Crocodylus siamensis* | NHMUK 1921.4.1.168 | Crocodylidae, Eusuchia |
| *Osteolaemus tetraspis* | FMNH 98936, NHMUK 1961-3-20-8, NHMUK 1962-6-30-5, NMS Z.2013.175, NMS Z.2014.3 | Crocodylidae, Eusuchia |
| *Mecistops cataphractus* | NHMUK 64.4.4.1, TMM M-3529 | Crocodylidae, Eusuchia |
| *Voay robustus* † | NHMUK PV R 36684, NHMUK PV R 36685 | Crocodylidae, Eusuchia |
| *Eosuchus lerichei* † | IRSNB-R-49; Delfino *et al*., 2005 | Gavialoidea, Eusuchia |
| *Gavialis gangeticus* | NHMUK 1935-6-4-1, NHMUK 1946-1-7-3, NHMUK 1996-7-7-4, NHMUK 2005-1601, TMM M-5490, UF-herp-118998 | Gavialidae, Eusuchia |
| *Tomistoma schlegelii* | NHMUK 1948-10-31-19, TMM M-6342, USNM 211322 | Gavialidae, Eusuchia |

**TABLE S3.** List of extinct non-thalattosuchian crocodylomorph specimens from the literature that lack palatal grooves.

| **Species** | **Literature** | **Taxonomic group** |
| --- | --- | --- |
| *Shartegosuchus asperapalatum* † | Dollman *et al*., 2018 | Shartegosuchoidea |
| *Caipirasuchus montealtensis* † | Andrade & Bertini, 2008b | Sphagesauridae, Notosuchia |
| *Sphagesaurus huenei* † | Pol, 2003 | Sphagesauridae, Notosuchia |
| *Yacarerani boliviensis* † | Novas *et al*., 2009 | Sphagesauridae, Notosuchia |
| *Campinasuchus dinizi* † | Carvalho *et al*., 2011 | Baurusuchidae, Notosuchia |
| *Baurusuchus salgadoensis* † | Carvalho *et al*., 2005 | Baurusuchidae, Notosuchia |
| *Kaprosuchus saharicus* † | Sereno & Larsson, 2009 | Mahajangasuchidae, Sebecia |
| *Hamadasuchus rebouli* † | Larsson & Sues, 2007 | Peirosauridae, Sebecia |
| *Montealtosuchus arrudacamposi* † | Carvalho *et al*., 2007 | Peirosauridae, Sebecia |
| *Calsoyasuchus valliceps* † | Tykoski *et al*., 2002 | Goniopholididae, Neosuchia |
| *Hulkepholis willetti* † | Salisbury & Naish, 2011 | Goniopholididae, Neosuchia |
| *Paluxysuchus newmanni* † | Adams, 2013 | Paluxysuchidae, Neosuchia |
| *Meridiosaurus vallisparadisi* † | Fortier *et al*., 2011 | Pholidosauridae, Neosuchia |
| *Oceanosuchus boecensis* † | Hua *et al*., 2007 | Pholidosauridae, Neosuchia |
| *Anthracosuchus balrogus* † | Hastings *et al*., 2015 | Dyrosauridae, Neosuchia |
| *Cerrejonisuchus improcerus* † | Hastings *et al*., 2010 | Dyrosauridae, Neosuchia |
| *Dyrosaurus maghribensis* † | Jouve *et al*., 2006 | Dyrosauridae, Neosuchia |
| *Guarinisuchus munizi* † | Barbosa *et al*., 2008 | Dyrosauridae, Neosuchia |
| *Isisfordia duncani* † | Salisbury *et al*., 2006 | Susisuchidae, Neosuchia |
| *Bernissartia fagesii* † | Martin *et al*., 2020 | Bernissartiidae, Neosuchia |
| *Rugosuchus nonganensis* † | Wu *et al*., 2001 | Paralligatoridae, Neosuchia |
| *Shamosuchus* spp. † | Turner, 2015 | Paralligatoridae, Neosuchia |
| *Allodaposuchus precedens* † | Delfino *et al*., 2008 | Allodaposuchidae, Eusuchia |
| *Lohuecosuchus megadontos* † | Narváez *et al*., 2015 | Allodaposuchidae, Eusuchia |
| *Hanyusuchus sinensis* † | Iijima *et al*., 2022 | Gavialodea, Eusuchia |
