# Supplementary figures and images for: Evidence for a novel cranial thermoregulatory pathway in thalattosuchian crocodylomorphs
Source: PeerJ. 2023 May 2;11:e15353. doi: 10.7717/peerj.15353 (PMC10162039; doi:10.7717/peerj.15353)

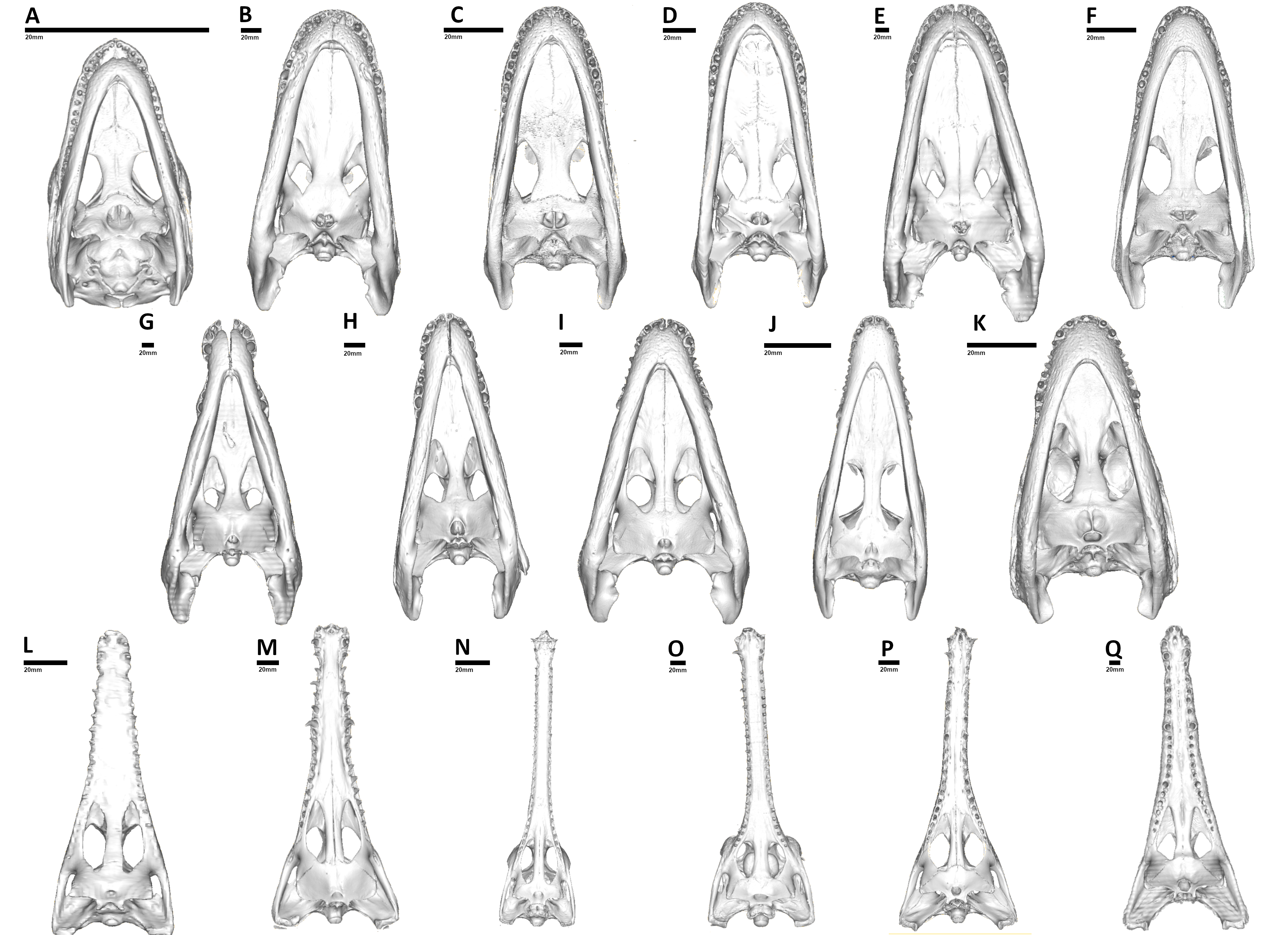

Supplement: Supplemental Information 2 — Note, none of the extant crocodylians have palatal grooves. (A) OUVC 10606, hatchling specimen of Alligator mississippiensis; (B) OUVC 9761, juvenile specimen of Alligator mississippiensis; (C) OUVC 11415, juvenile specimen of Alligator mississippiensis; (D) TMM M-983, juvenile specimen of Alligator mississippiensis; (E) USNM 211233, adult specimen of Alligator mississippiensis; (F) FMNH 73711, subadult specimen of Caiman crocodilus; (G) FMNH 59071, adult specimen of Crocodylus acutus; (H) MNB AB50.071, adult specimen of Crocodylus rhombifer; (I) TMM M-4980, adult specimen of Crocodylus moreletii; (J) OUVC 10899, juvenile specimen of Crocodylus porosus; (K) FMNH 98936, subadult specimen of Osteolaemus tetraspis; (L) TMM M-6807, subadult specimen of Crocodylus johnstoni; (M) TMM M-3529, adult specimen of Mecistops cataphractus; (N) TMM M-5490, subadult specimen of Gavialis gangeticus; (O) UF herp 118998, adult specimen of Gavialis gangeticus; (P) TMM M-6342, subadult specimen of Tomistoma schlegelii; (Q) USNM 211322, adult specimen of Tomistoma schlegelii. [file peerj-11-15353-s002.png]

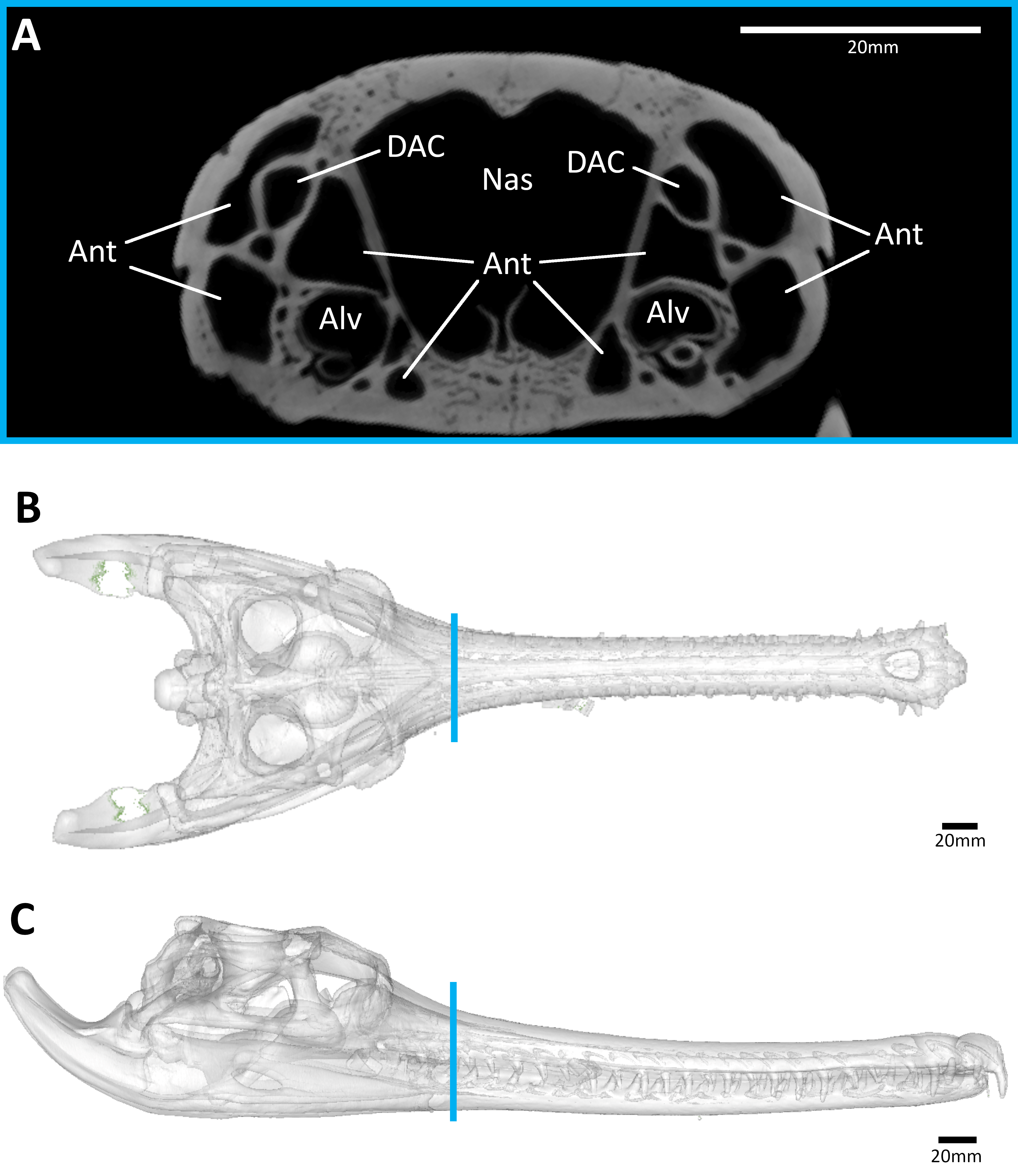

Supplement: Supplemental Information 3 — (A) snout coronal view showing the lack of palatal canals. Three-dimensional reconstruction of the skull in (B) dorsal, and (C) lateral view, both showing the palatal canals in red and the CT slice of (A) shown in blue. Abbreviations: Ant, antorbital pneumatic sinus; Alv, alveolus; DAC, dorsal alveolar canal; Nas, nasal cavity. [file peerj-11-15353-s003.png]

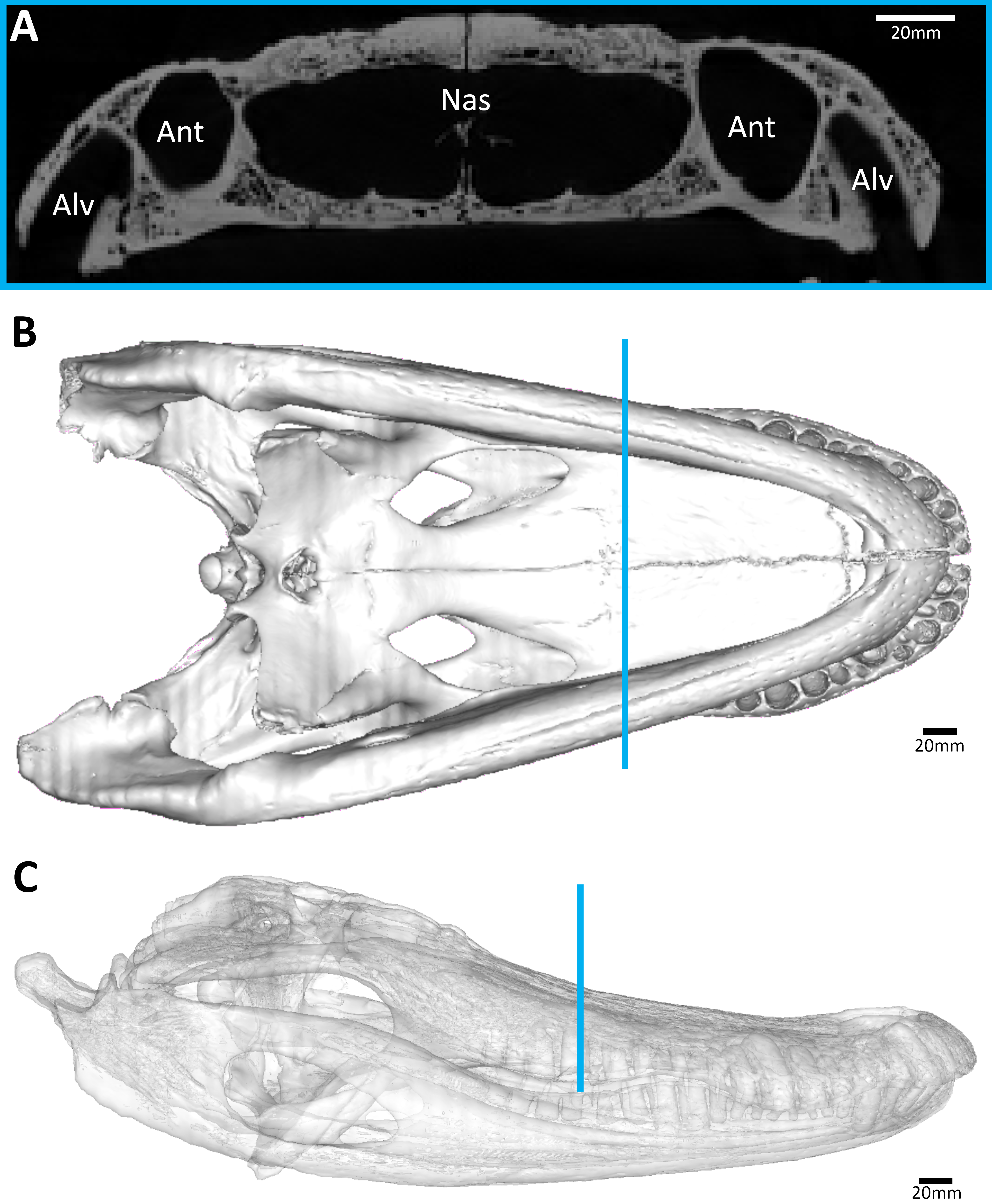

Supplement: Supplemental Information 4 — (A) snout coronal view showing the lack of palatal canals. Three-dimensional reconstruction of the skull in (B) dorsal, and (C) lateral view, both showing the palatal canals in red and the CT slice of (A) shown in blue. Abbreviations: Ant, antorbital pneumatic sinus; Alv, alveolus; Nas, nasal cavity. [file peerj-11-15353-s004.png]

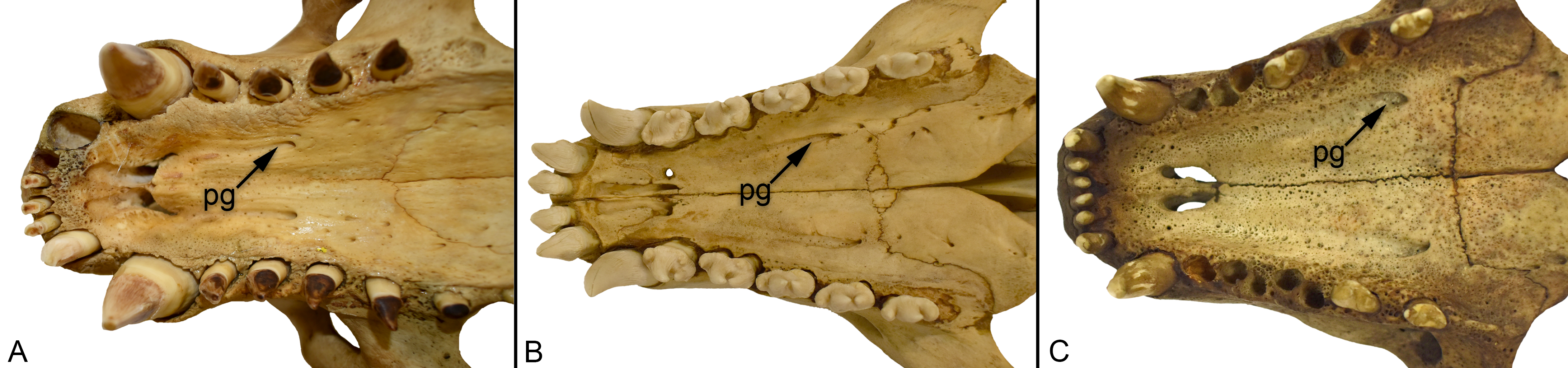

Supplement: Supplemental Information 5 — (A) the Antarctic fur seal (Arctocephalus gazella) NMS 2007.91.10; (B) the Leopard seal (Hydrurga leptonyx) NMS 1822.240.T29; (C) the Harbour seal (Phoca vitulina) NMS 1996.99.13. (A) is an otariid, while (B) and (C) are phocids. Abbreviations: PG, palatal groove. [file peerj-11-15353-s005.png]

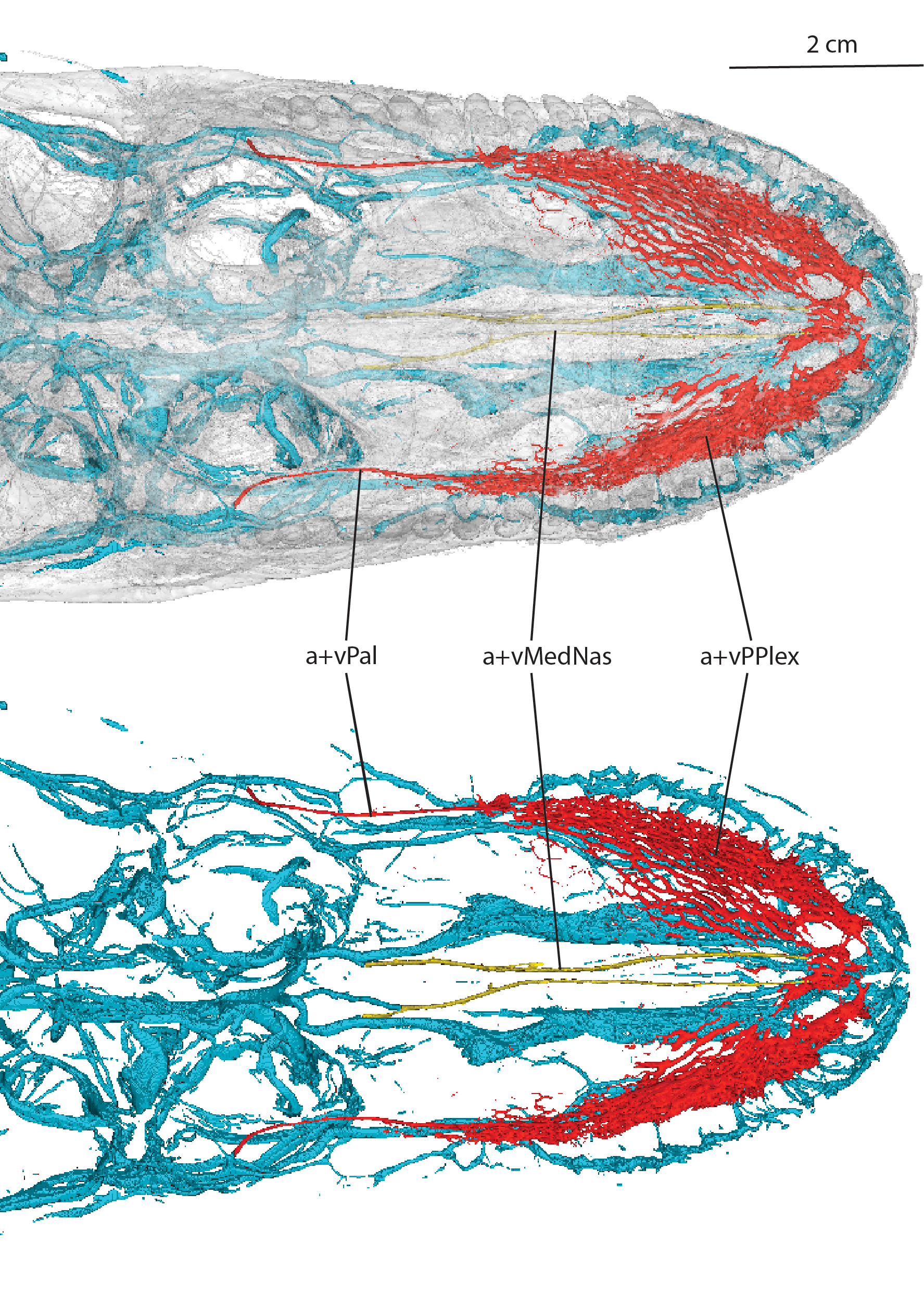

Supplement: Supplemental Information 6 — Cephalic vasculature with the medial nasal artery/vein shown in yellow and the palatine artery/vein and palatal plexus shown in red, (A) with the transparent skull, and (B) just the vasculature. Abbreviations: a+vMedNas, medial nasal artery and vein; a+vPal, palatine artery and vein; a+vPPlex, arterial and venous palatal plexus. [file peerj-11-15353-s006.png]

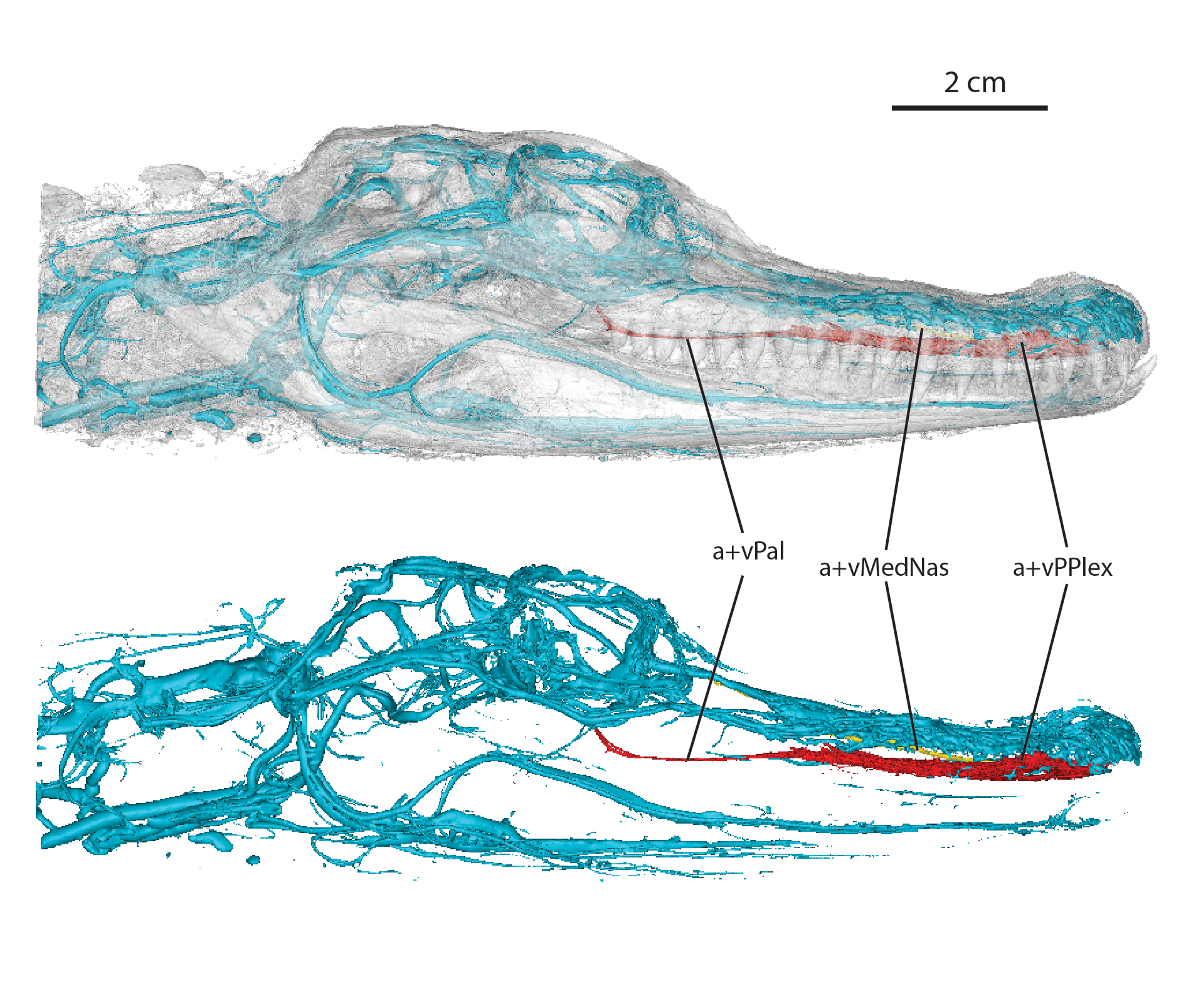

Supplement: Supplemental Information 7 — Cephalic vasculature with the medial nasal artery/vein shown in yellow and the palatine artery/vein and palatal plexus shown in red, (A) with the transparent skull, and (B) just the vasculature. Abbreviations: a+vMedNas, medial nasal artery and vein; a+vPal, palatine artery and vein; a+vPPlex, arterial and venous palatal plexus. [file peerj-11-15353-s007.png]
